# Exploring *Bifidobacterium* species community and functional variations with human gut microbiome structure and health beyond infancy

**DOI:** 10.20517/mrr.2023.01

**Published:** 2023-03-31

**Authors:** Ruben Ladeira, Julien Tap, Muriel Derrien

**Affiliations:** ^1^Advanced Health & Science, Danone Global Research & Innovation Center, Gif-sur-Yvette 91190, France.; ^2^Université Paris-Saclay, INRAE, AgroParisTech, Micalis Institute, Jouy-en-Josas 78350, France.

**Keywords:** Human gut microbiome, *Bifidobacterium longum*, *Bifidobacterium adolescentis*, partitions, function, MAGs, health

## Abstract

**Aim:** The human gut *Bifidobacterium* community has been studied in detail in infants and following dietary interventions in adults. However, the variability of the distribution of *Bifidobacterium* species and intra-species functions have been little studied, particularly beyond infancy. Here, we explore the ecology of *Bifidobacterium* communities in a large public dataset of human gut metagenomes, mostly corresponding to adults.

**Methods:** We selected 9.515 unique gut metagenomes from curatedMetagenomicData. Samples were partitioned by applying Dirichlet’s multinomial mixture to *Bifidobacterium* species. A functional analysis was performed on > 2.000 human-associated *Bifidobacterium* metagenome-assembled genomes (MAGs) paired with participant gut microbiome and health features.

**Results:** We identified several *Bifidobacterium*-based partitions in the human gut microbiome differing in terms of the presence and abundance of *Bifidobacterium* species. The partitions enriched in both *B. longum* and *B. adolescentis* were associated with gut microbiome diversity and a higher abundance of butyrate producers and were more prevalent in healthy individuals. *B. bifidum* MAGs harboring a set of genes potentially related to phages were more prevalent in partitions associated with a lower gut microbiome diversity and were genetically more closely related.

**Conclusion:** This study expands our knowledge of the ecology and variability of the *Bifidobacterium* community, particularly in adults, and its specific association with the gut microbiota and health. Its findings may guide the rational selection of *Bifidobacterium* strains for gut microbiome complementation according to the individual’s endogenous *Bifidobacterium* community. Our results also suggest that gut microbiome stratification for particular genera may be relevant for studies of variations of species and associations with the gut microbiome and health.

## INTRODUCTION

*Bifidobacterium* is a gut microbiome component, the abundance of which varies with age and health status. Many studies have shown that various metabolic, immune, and intestinal disease states coincide with *Bifidobacterium* depletion from the human gut microbiota^[1]^. *Bifidobacterium* species metabolize a wide range of simple and complex glycans, some dietary and others host-derived^[2]^, and produce various metabolites, such as organic acids, B vitamins^[3]^, tryptophan-derived metabolites^[4]^, and neurotransmitters, such as GABA^[5]^. As such, *Bifidobacterium* performs a number of roles in interactions between the gut microbiota and the host. The prevalence of *Bifidobacterium* species varies considerably during the lifespan of an individual human, particularly between infancy and adulthood. The abundance and prevalence of *B. bifidum, B. breve, B. longum subsp. Longum,* are typically high in the infant gut microbiota across different populations with variation for *B. longum subsp. infantis*^[6-8]^. The prevalence of *Bifidobacterium* generally exceeds 90% in healthy adults, with just a few species present per subject^[9]^, mostly *B. adolescentis* and *B. longum subsp longum* (*B. longum*), which are able to metabolize complex dietary carbohydrates^[10]^. The *Bifidobacterium* content of the human gut microbiome has been well studied in both healthy and diseased individuals and following dietary interventions, but little is known about the variability of *Bifidobacterium* species between subjects and its effects on gut microbiome composition, function, and human health. Most cross-sectional or interventional studies performed to date have had a limited sample size, with potentially restricted variation in the gut microbiome, including that for *Bifidobacterium* communities. Over the last decade, large cross-sectional cohorts (> 1,000 subjects) have been established and studied to disentangle the specific associations between intrinsic and extrinsic factors and the gut microbiome. For instance, the abundance of *Bifidobacterium* has been shown to depend on genetics, specifically lactase persistence/non-persistence^[11]^, and dietary factors, such as carbohydrates^[11,12]^. Despite their significant relevance for identifying the major factors underlying gut microbiome variation, some single cohorts may display a lack of gut microbiome variation as a function of health status, lifestyle, age, and taxonomic and functional resolution.

The use of public databases containing large amounts of human gut microbiota shotgun metagenomic data spanning different ages, countries, health statuses, and lifestyles has greatly increased in recent years, providing new insight into the association of the gut microbiota with the host, environmental factors, and the reconstruction of metagenome-assembled genomes (MAGs)^[13-18]^. An extensive study by Pasolli *et al.* yielded over 150.000 MAGs^[16]^. Large-scale analyses of targeted bacterial species have improved our understanding of their diversity, ecology, and association with health and lifestyle. For instance, the study of *Faecalibacterium prausnitzii*^[19]^, *Akkermansia muciniphila*^[20]^, and *Prevotella copri*^[21]^ has revealed new diversity and specific functional features associated with the host and environmental factors. Despite the increasing availability of metagenomic data, we still know very little about the metabolic contributions of strains within an ecological niche. Strains of *Bifidobacterium* have long been considered of major interest for use as probiotics^[22,23]^, but the variability and ecology of resident *Bifidobacterium* species and their association with the host and the gut microbiome have been little explored in large-scale studies. One recent study characterized the strain dynamics, pangenome, and genomic diversity of the main *Bifidobacterium* species from the human gut in early life with MAGs^[6]^. However, functional pangenomic analyses of *Bifidobacterium* in the adult human gut are lacking.

We performed an exploratory analysis of the ecology of *Bifidobacterium* based on data from a public database containing human gut microbiome data, mostly for adult subjects. We first confirmed the previously reported associations with health, age, and other factors. We then identified *Bifidobacterium* partitions of the gut differing in terms of the abundance of *Bifidobacterium*, species composition, gut microbiome features, and health status. Finally, using MAG-based pangenomic analysis, we showed that the prevalence of some functional features of some *Bifidobacterium* species differed between health-associated *Bifidobacterium* partitions. This study paves the way for more precise approaches to guide the selection of *Bifidobacterium* strains for gut microbiome complementation in adulthood and, ultimately, human health.

## METHODS

### Pooled metagenomic studies dataset

We extracted taxonomic data from the curatedMetagenomicData (cMD) R package (Pasolli *et al.*) (version 3.0, release 2021), which consists of manually curated metadata together with all the taxonomic read counts aggregated per species with MetaPhlAn3, for 86 studies (17,959 samples). Gut metagenomes with more than five million reads were retained, and one duplicate study (referred to as “LeChatelierE_2013”) was excluded. The read counts for the samples were sum-collapsed by genus. The resulting feature table was rarefied to a depth of 1.000.000 counts per sample for alpha diversity analysis. Filtering for origin (stools), with the selection of one fecal sample per subject (highest number of reads), resulted in 9,515 unique samples (61 studies). This dataset was used for a global description of the abundance of *Bifidobacterium* and the prevalence of *Bifidobacterium* species across different metadata curated in cMD: age, lifestyle, antibiotic use status, and health status.

The age categories were as follows: newborn (< 1 year of age), child (age ≥ 1 year and < 12 years), school-age individuals (age ≥ 12 and < 19 years); adult (age ≥ 19 years), senior (> 65 years).

Lifestyle was classified as westernized or non-westernized, and antibiotic use was classified as yes (the month preceding stool sample collection) or no.

Health-related metadata were aggregated into six categories as follows: *control*: subject known to be healthy; *adenoma*: patients with all types and subtypes of adenoma; *colorectal*: patients with colorectal cancers including metastases; *metabolic*: patients with metabolic conditions including atherosclerotic cardiovascular disease, hypercholesterolemia, hypertension, type 2 diabetes, and impaired glucose tolerance; *bowel*: patients with inflammatory bowel disease (IBD); *arthritis*: patients with rheumatoid arthritis or Behçet’s disease (BD).

### *Bifidobacterium*-based clustering of the gut microbiome

Samples were partitioned by applying Dirichlet’s Multinomial Mixture (DMM) modeling to the microbiota data^[24]^ for 32 detected *Bifidobacterium* species with counts across cMD. We filtered the 9.515 datasets as follows to obtain a final dataset relating to 5.329 subjects for DMM: (1) We retained individuals who had not had antibiotic treatment as declared in the cMD (antibiotic use = no) (*N* = 216) or without information (*N* = 3.571) to prevent bias in the diversity calculation; (2) We excluded subjects with a total count < 500, to overcome *Bifidobacterium* underdetection issues (*N* = 19); (3) We excluded subjects with no *Bifidobacterium* species total reads count as a DMM standard (*N* = 380).

DMM models were calculated for different numbers (k) of clusters, k ∈ [1,30], and evaluated with the Bayesian information criterion (BIC) and five different seeds. These methods are based on minimizing a penalized criterion, taking into account model fit and complexity. We chose three random seeds and calculated the minimum k for different model fits, and then selected the most frequently observed. We determined the contribution of each *Bifidobacterium* species to each DMM cluster from the calculated models.

### Functional analysis of *Bifidobacterium* MAGs

We retrieved 3,973 metagenomic-assembled genomes (MAGs) assigned to 15 *Bifidobacterium* species from http://opendata.lifebit.ai/table/?project=SGB. The MAGs were previously decontaminated and taxonomically assigned by Mash^[16]^. The study identifier, sample identifier, assigned species, and completeness were collected for each MAG. Prodigal was used for gene calling for each MAG, and more than six million genes were called. The computation time required for annotation was decreased by clustering the MAG gene against a non-redundant gut *Bifidobacterium* gene catalog, using CD-HIT at 95% nucleotide identity, with a minimum sequence overlap of 90%. Non-redundant *Bifidobacterium* genes were annotated with EggNOG 5.0^[25]^ and dbCAN^[26]^ version 3 to obtain orthologous genes (OGs) and CAZy families, respectively. Quality was ensured by selecting the MAGs with completeness > 80%. *Bifidobacterium* species with at least 30 associated MAGs were selected, giving a total of six species. 820 MAGs were assigned to *B. longum*, 700 to *B. adolescentis*, 339 to *B. bifidum*, 178 to *B. pseudocatenulatum*, 54 to *B. catenulatum*, and 34 to *B. dentium*. Pairwise distances were calculated for all MAGS within each species, with Mash v2.347 and the default sketch size. Hierarchical clustering was then performed for each species with the “ward.D2” method and the “pheatmap” R package (1.0.12).

### Statistical analysis

The associations between *Bifidobacterium* partitions and quantitative variables (notably α-diversity and *Bifidobacterium* abundances) were analyzed with Kruskal-Wallis tests and a post-hoc test (Mann-Whitney test, adjusted for FDR). Pearson’s chi-squared test was used to determine whether 1) *Bifidobacterium* partitions were associated with categorical variables (age, lifestyle, health status) and 2) whether the prevalence of OGs in MAGs for each species was associated with health-associated partitions, adjusted for FDR (within species). DESeq2 (v1.28.1) was used to identify bacterial species for which abundance differed between *Bifidobacterium* partitions with the “poscounts” normalization option to accommodate the sparsity of microbiota data. The global effects of the *Bifidobacterium* were estimated in likelihood ratio tests and Wald tests for pairwise comparisons of clusters. A FDR correction for multiple testing was applied to each test to account for the number of species tested. Log_2_ fold-changes in expression are expressed as the estimate ± standard error. When specified, FDR corrections were applied with the Benjamini-Hochberg procedure.

## RESULTS

### Analysis of pooled metagenomic studies recapitulates major findings of human gut *Bifidobacterium* ecology

We used the “curatedMetagenomicData” (cMD, version 3) database (Pasolli *et al.*) to study the ecology of the *Bifidobacterium* community in the human gut microbiome. The cMD provides standardized, curated human microbiome data with several pieces of metadata per participant. We selected only gut metagenomes (one per subject) and obtained 9.515 unique samples [Table 1].

**Table 1:** Datasets used in the study

	**All individuals**	**No antibiotic intake**	**No antibiotic intake** **and adults only**
**Characteristic**	***N* = 9.515**	***N* = 5.728**	***N* = 4.921**
**Antibiotic intake**	216 (3.6%)		
Unknown	3.571		
No		5.728 (100%)	4.921 (100%)
**Health status**			
Control	7.016 (79%)	4.876 (85%)	4.348 (88%)
Adenoma	153 (1.7%)	39 (0.7%)	29 (0.6%)
Colorectal	439 (4.9%)	110 (1.9%)	74 (1.5%)
Metabolic	656 (7.4%)	495 (8.6%)	320 (6.5%)
Bowel	535 (6.0%)	99 (1.7%)	45 (0.9%)
Arthritis	94 (1.1%)	89 (1.6%)	85 (1.7%)
Behçet’s disease (BD)	20 (0.2%)	20 (0.3%)	20 (0.4%)
Unknown	602		
**Age category**			
Newborn	278 (2.9%)	137 (2.4%)	
Child	322 (3.4%)	160 (2.8%)	
School-age	135 (1.4%)	88 (1.5%)	
Adult	7.745 (81%)	4.921 (86%)	4.921 (100%)
Senior	1,035 (11%)	422 (7.4%)	
**Westernized lifestyle**	8.701 (91%)	5.577 (97%)	4.774 (97%)
*n* (%)			

This dataset contains predominantly data for adults with a westernized lifestyle. Individuals under the age of 19 years accounted for less than 8% of this dataset [newborns (2.9%), children (3.4%), and school-age (1.4%)] [Table 1 and Supplementary Figure 1].

Given the multiple differences in analytical procedures between the studies included in the cMD, we first investigated whether our analysis of the cMD database could reproduce published findings for *Bifidobacterium* in humans, such as differences according to age, lifestyle, antibiotic use, and health status.

We found that subjects with a westernized lifestyle had higher relative abundances of *Bifidobacterium* (Mann-Whitney, *P* < 0.001) [Supplementary Figure 2] and a higher prevalence of *B. animalis* and *B. longum* [Supplementary Figure 3]. The gut microbiome of newborns was more enriched in *Bifidobacterium* (median of 7.76 %, IQR 0.48%-41.3%) than that of the other age categories (median 2%, IQR 0.01%-9.8%) (Kruskal Wallis, *P* < 0.001) [Supplementary Figure 2], with a higher prevalence of *B. breve* in children under the age of three years (50%), decreasing to < 10% thereafter. *B. adolescentis* was more prevalent in adults (71%) than in younger subjects (35%-50%) and seniors (60%). By contrast, *B. longum* was highly prevalent at all ages (> 80%) [Supplementary Figure 3]. The relative abundance of *Bifidobacterium* was significantly lower in most of the disease groups, especially Bowel (IBD) (median 0.03%, IQR 0%-0.2%) than in healthy individuals (median 3.4%, IQR 0.6%-10.7%) (Kruskal Wallis, *P* < 0.001) [Supplementary Figure 2]. In particular, *B. adolescentis* was more prevalent in healthy individuals (70%) than in those with the disease, particularly for IBD and metabolic diseases (40%-50%), whereas the opposite pattern was found for *B. dentium* [Supplementary Figure 3]. Finally, the relative abundance of *Bifidobacterium* was lower in subjects with recent antibiotic intake (Mann-Whitney, *P* < 0.001) [Supplementary Figure 2]. Despite the considerable analytical differences between studies, this dataset reproduced the major associations previously reported between *Bifidobacterium* and age^[27]^, lifestyle^[28]^, health status^[1,29,30]^, and antibiotic use^[31-33]^ in individual cohorts.

### *Bifidobacterium*-based partitioning of the human gut microbiome

**Figure 1:**
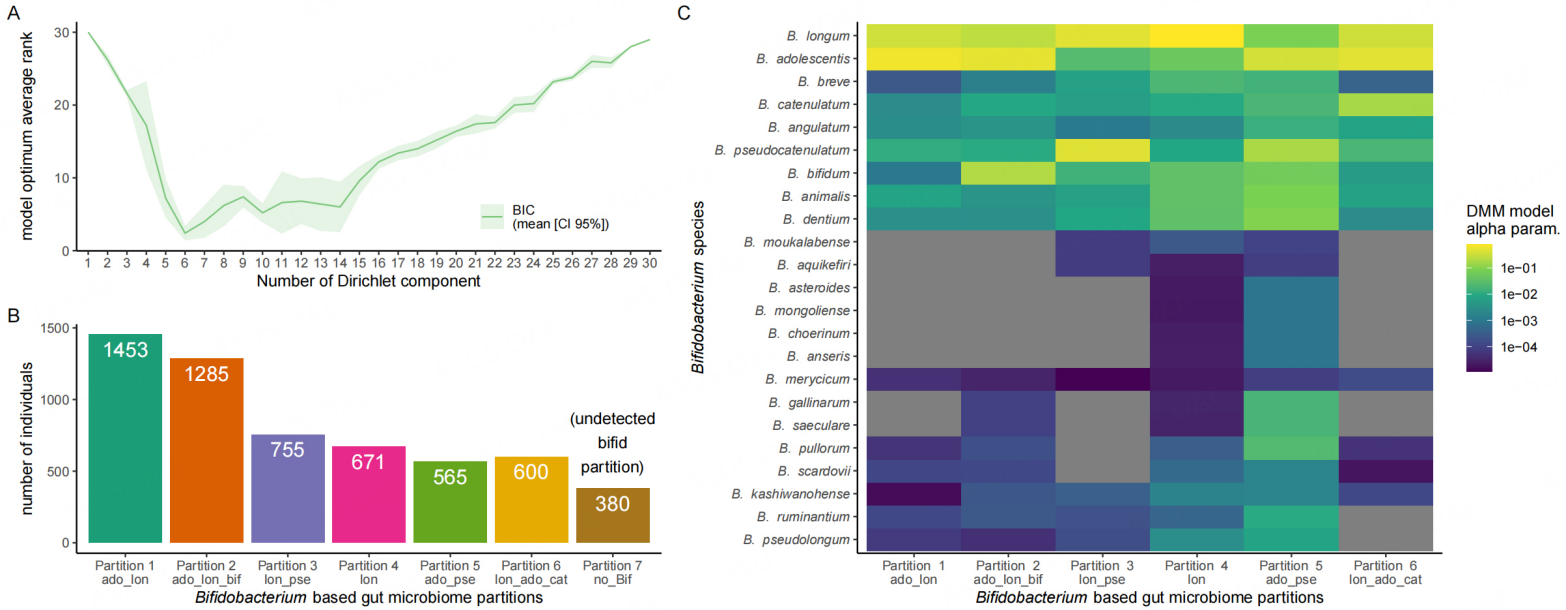
*Bifidobacterium*-based gut microbiome partitions. (A) Model fit according to BIC; (B) distribution of subjects across the 7 partitions. Partition #7 consists of subjects for whom no *Bifidobacterium* was detected (*no-Bif*); (C) scaled contribution of each *Bifidobacterium* species to each partition. Higher DMM model contributions are associated with a higher relative abundance of a particular species. Species are ordered according to hierarchical clustering based on Euclidean distance. Gray indicates alpha parameters below 10^-5^. More abundant species: *B. longum* to *B. dentium* and sub-dominant species: *B. moukalabense* to *B. pseudolongum*.

We further explored the ecology and variation of *Bifidobacterium* species between subjects. We used the Dirichlet multinomial mixtures (DMM) partitioning method^[24]^, which is commonly used to identify partitions of the human gut microbiome; however, in this case, we applied it exclusively to *Bifidobacterium* species. This made it possible to focus specifically on the association of the variable within-*Bifidobacterium* community distribution with the ecological features of the gut microbiome, lifestyle, and health. We applied DMM to 5.329 subjects (see methods). On the basis of BIC minimization, k = 6 was chosen for individuals for whom *Bifidobacterium* was detected [Figure 1A]. We added a group (k = 7) corresponding to subjects for whom no *Bifidobacterium* reads were detected (*n* = 380). All partitions had a median number of reads above 30 M [Supplementary Table 1].

Partitions #1 and #2 accounted for 48% of subjects, whereas the partition corresponding to the detection of no *Bifidobacterium* species (partition #7) accounted for the smallest number of subjects (17%) [Figure 1B]. We extracted the scaled contribution of each *Bifidobacterium* species to each partition, which reflects the relative abundance of these species within *Bifidobacterium*. Some of the dominant *Bifidobacterium* species (shown in yellow) differed in abundance between partitions [Figure 1C].

In partitions #1, #2, and #6, both *B. adolescentis* and *B. longum* were abundant, whereas in partitions #3, #4, and #5, either *B. adolescentis* or *B. longum* was the dominant species. Partition #1 consisted mostly of *B. adolescentis* and *B. longum (ado_lon),* whereas partition #2 also included *B. bifidum* (*ado_lon _bif*), and partition #6 included *B. catenulatum (lon_ado_cat).* Partition #3 composition was dominated by both *B. longum and B. pseudocatneulatum (lon_pse*), whereas partition #4 consisted mostly of *B. longum (lon).* Partition #5 was dominated by both *B. adolescentis* and *pseudocatneulatum* (*ado_pse*) and contained multiple sub-dominant species. For the less abundant species, *B. breve*, *B. animalis,* and *B. dentium,* relative abundance was highest in partitions #4 *(lon)* and #5 (*ado_pse)*.

### Association between *Bifidobacterium*-based partitions and the gut microbiome and health

**Figure 2:**
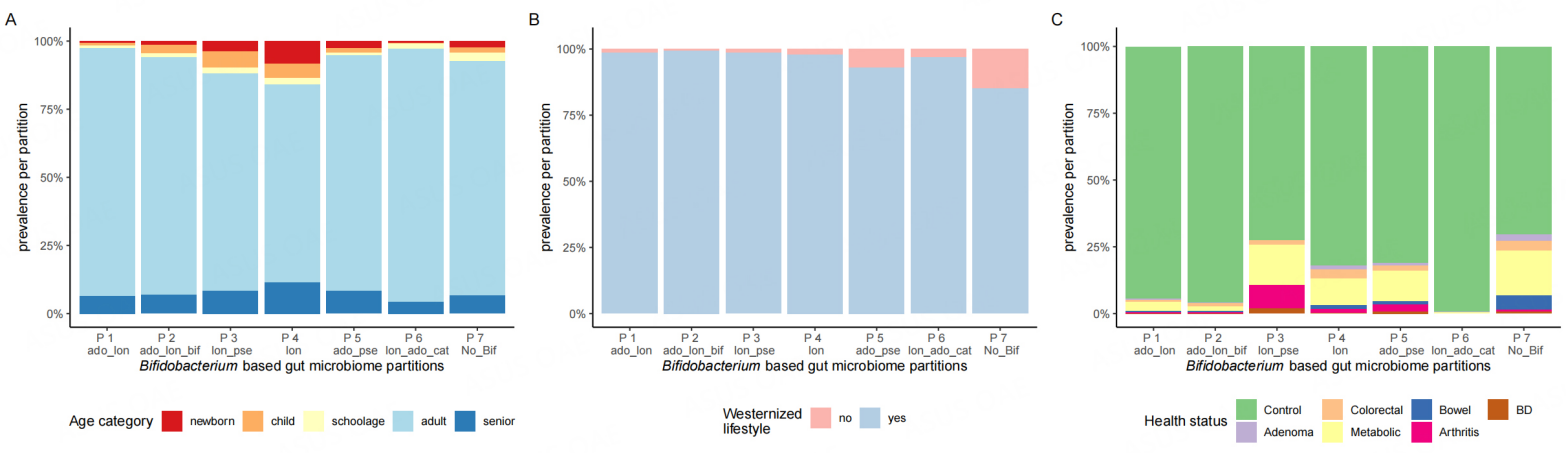
Distribution of subjects between *Bifidobacterium*-based gut microbiome partitions according to metadata (without recent antibiotic intake). (A) Age category; (B) westernized lifestyle (adults); (C) health status (adults).

We then investigated the distribution of *Bifidobacterium*-based partitions as a function of age category, lifestyle (westernized/non-westernized), and health conditions [Figure 2A-C and Supplementary Figure 4]. The associations of *Bifidobacterium* partitions with health status (healthy or with one of the health conditions considered), lifestyle (westernized/ non-westernized), and age category were significant (chi-squared, *P*-value < 0.05).

The prevalence of seniors and subjects with a non-western lifestyle was higher in the *lon* and *no_Bif* partitions, respectively [Figure 2C]. The *Bifidobacterium* partitions *lon_pse*, *lon, ado_pse*, and *no_Bif* included ~70% healthy subjects, whereas more than 90% of the subjects in the *Bifidobacterium* partitions *ado_lon*, *ado_lon_bif*, and *lon_ado_cat* were healthy. We also found that 64% of the healthy subjects belonged to the *Bifidobacterium* partitions *ado_lon*, *ado_lon_bif,* and *lon_ado_cat*, whereas 74% of the subjects with health conditions belonged to the *Bifidobacterium* partitions *lon_pse, lon, ado_pse*, and *no_Bif*.

**Figure 3:**
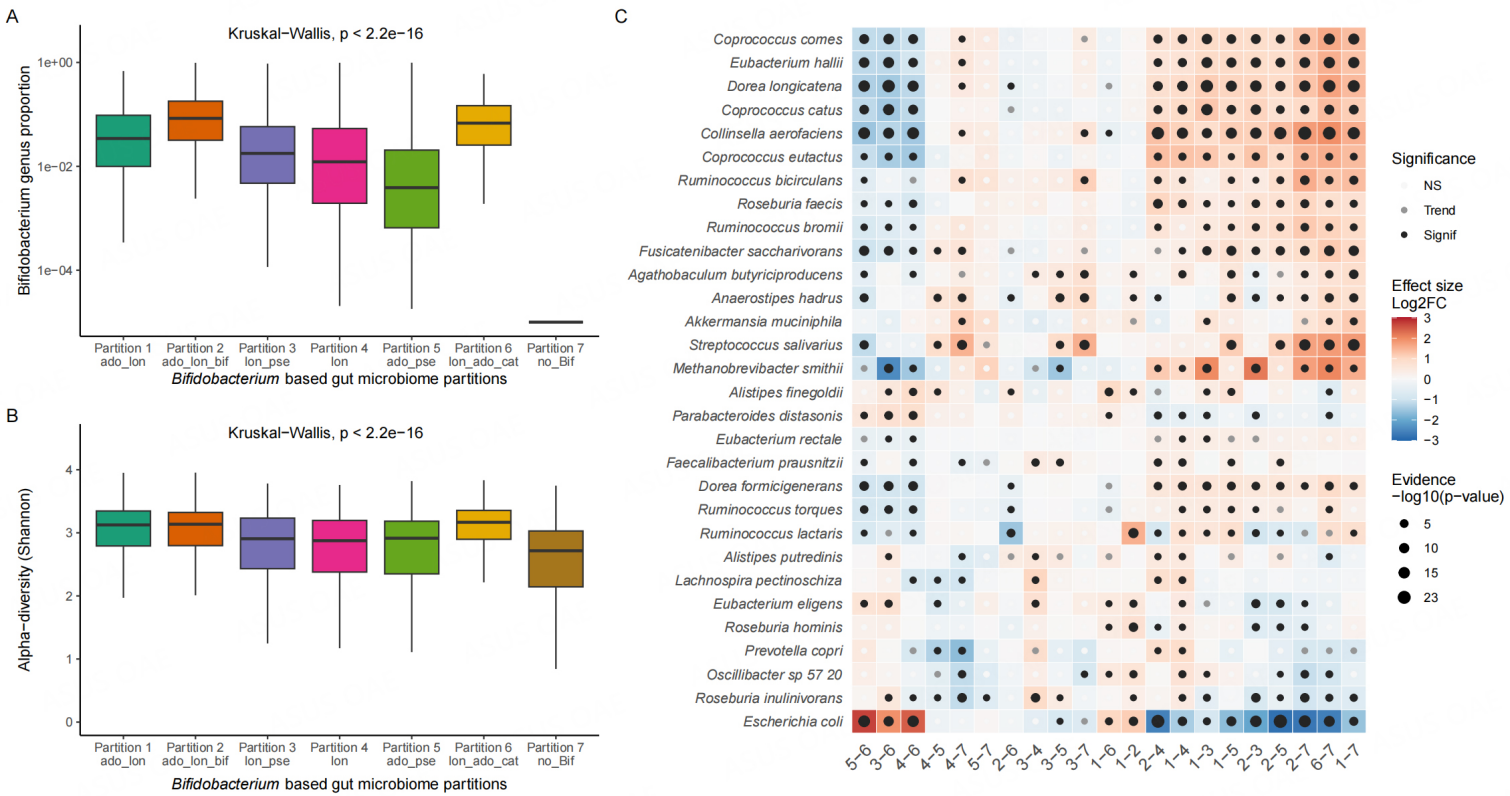
Variation of the gut microbiome between *Bifidobacterium*-based partitions. (A) Relative abundance of *Bifidobacterium* across partitions; (B) species-based Shannon index for the gut microbiome; (C) differential analysis of the gut microbiome across partitions. The top 30 most abundant species are depicted. Red indicates a higher species abundance in the first partition tested (significant when FDR < 0.05, trend when FDR < 0.1). Species and partitions are ranked according to hierarchical clustering based on Euclidean distance, with the exclusion of *Bifidobacterium* species from the graph.

We then investigated the association of the *Bifidobacterium* partitions with the gut microbiome. We found that *Bifidobacterium* partitions were significantly associated with *Bifidobacterium* abundance [Figure 3A]. and gut microbiome Shannon diversity [Figure 3B] (Kruskal-Wallis, *P* < 0.001). *Bifidobacterium* partitions *ado_lon*, *ado_lon_bif*, and *lon_ado_cat* had a higher relative abundance of *Bifidobacterium*, and a higher gut microbiome α-diversity (Shannon index) than the other partitions [Supplementary Table 1] (Mann-Whitney test, *P* < 0.001). Given that cMD also includes infants less than one-year-old, which may influence alpha-diversity results, we further investigated whether partitions could be detected in single adult cohorts in which samples were processed with the same analytical procedure. We selected three large cohorts from the cMD (> 1.000 adults) and extracted their *Bifidobacterium* partitions: (1) 1.098 individuals from the UK enrolled in the Personalised Responses to Dietary Composition Trial (PREDICT 1) study^[12]^; (2) 1.135 participants from the Dutch population-based cohort LifeLines-DEEP^[18]^; and (3) 800 individuals from an Israeli cohort. All the identified partitions were detected in the three cohorts with different prevalences [Supplementary Figure 5]. As for the cMD, the *ado_lon* and a*do_lon_bif* partitions were the most prevalent (> 50%), and the *no_Bif* partition was the least prevalent (< 10%). We identified several partitions related to *Bifidobacterium* composition in the adult gut microbiome. These differences were also observed when only adults from the cMD were selected [Supplementary Table 1].

We then used DESeq2 to identify bacterial species for which abundance differed between partitions (FDR < 0.05, Wald test) [Figure 3C]. We found that *Bifidobacterium* partitions enriched in both *B. longum* and *B. adolescentis* (*ado_lon*, *ado_lon_bif*, and *lon_ado_cat)* shared common, differentially abundant bacterial species compared to other partitions (contrasts on the left and right parts of the heatmap). The *ado_lon*, *ado_lon_bif*, and *lon_ado_cat* partitions had a lower abundance of *E. coli* and a higher abundance of several butyrate producers (*Roseburia faecis, Coprococcus catus, C. eutactus, C. comes,* and *Eubacterium hallii*). The *no_Bif* partition was associated with a lower abundance of *Streptococcus salivarius* than the other *Bifidobacterium*-based partitions.

Overall, the partitions enriched in both *B. longum* and *B. adolescentis* were associated with higher gut microbiome diversity and abundance of butyrate producers and were more prevalent in healthy individuals (i.e., health-associated *Bifidobacterium* communities).

### A pangenomic analysis of *Bifidobacterium* reveals functions associated with gut ecology and health

Finally, we investigated whether intra-species functions were associated with *Bifidobacterium* partitions as a surrogate for a more diverse gut microbiome and a higher prevalence of healthy subjects. We selected 2,263 MAGs constructed from an extensive dataset^[16]^ included in the cMD database. Therefore, we could pair participants’ *Bifidobacterium* partitions with their *Bifidobacterium* MAG content. An analysis of 11,673 unique OGs functionally distinguished MAGs from different *Bifidobacterium* species on the basis of OGs prevalence (chi-squared test, FDR < 0.05 within species) [Supplementary Table 2]. The most significant OGs were that for asparagine synthase (COG0367), which was detected in 99% of the MAGs assigned to *B. adolescentis* and 0.25% of those from other species. We confirmed known functional differences, relating, for example, to glycoside hydrolases (GH) involved in the metabolism of host carbohydrates (mucin/milk), such as GH 20, GH 29, GH 33, and GH 95, which were specific to *B. bifidum* and had prevalences ranging from 90 to 97%, *vs.* 0.1% in other species. Similarly, we detected a high prevalence of alpha-L-arabinofuranosidase (COG3534) for the MAGs *of B. longum* (99% *vs.* 0.7% in other species). For *B. pseudocatenulatum*, we detected OGs assigned to the GH 43 family (xylosidase).

**Figure 4:**
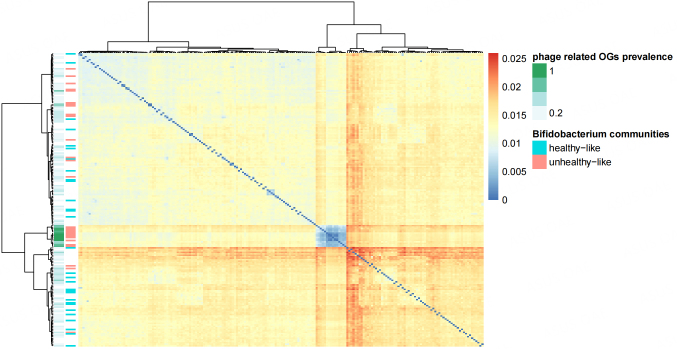
Heatmap of Mash distances between the *B. bifidum* MAGs. The heatmap is annotated with the prevalence of the 15 OGs related to phages. The gradient from blue to red indicates increasing Mash genetic distances between the MAGs. Hierarchical clustering was performed by the Ward2 method. MAGs derived from subjects without *Bifidobacterium* partition information are shown in white.

We then studied the functions of *Bifidobacterium* associated with previously identified *Bifidobacterium* partitions (health-associated *ado_lon*, *ado_lon_bif*, and *lon_ado_cat* or others *(lon_pse*, *lon, ado_pse*, and *no_Bif*) by pairing MAG content to a *Bifidobacterium* partition for each subject. This analysis compared the gene content of MAGs from specific *Bifidobacterium* species regardless of their differential abundance between health groups. We found 38 OGs significantly associated with these two types of *Bifidobacterium* partitions (chi-squared, FDR < 0.1). Fifteen of these OGs were less prevalent in health-associated *Bifidobacterium* partitions (< or > 25% prevalence in health-associated and others, respectively) [Supplementary Table 3]. These OGs were assigned to phages and included integrases, transposases, and helicases. We then investigated the phylogenetic relationships between the MAGs harboring these 15 OGs in *B. bifidum*. We computed Mash distances between 198 *B. bifidum* MAGs and visualized the prevalence of 15 significant OGs in the 198 *B. bifidum* MAGs. These 15 OGs had a higher prevalence in a cluster of MAGs associated with the *Bifidobacterium* partitions most frequently detected in individuals with diseases [Figure 4]. This finding suggests that a *B. bifidum* subspecies or strain may be enriched in phage-related genes in subjects with more altered gut microbiomes.

## DISCUSSION

In this study, we performed a large-scale analysis of the *Bifidobacterium* community in the human gut microbiome, with data from a large adult population, including individuals with various health conditions. By combining ecological and functional analyses of the *Bifidobacterium* community, we identified variable associations of partitions of *Bifidobacterium* species and functions associated with gut microbiome features and human health. Overall, our results confirm and extend previous findings on the ecological and functional relevance of the *Bifidobacterium* community for the gut microbiome and human health.

The human gut microbiome varies significantly between subjects, and this variation may obscure the effect of diet or treatment. Stratification of the gut microbiome on the basis of its composition has been used to identify the subjects most likely to respond to dietary interventions^[34]^ or medical treatments^[35]^. *Bifidobacterium* is a common member of the human gut microbiome, with different species co-existing in the host at different ages^[36]^. A number of studies have shown that various metabolic, immune, and intestinal disease states coincide with the depletion of *Bifidobacterium* from the gut microbiota^[1]^. Here, we studied the variability of the gut microbiome as a function of the resident *Bifidobacterium* community, using a public database compiling curated metagenomics-based studies, mostly performed in adults from Western countries. We first checked that we could reproduce the previously reported findings of a lower abundance of *Bifidobacterium* species in antibiotic users^[31-33]^ and individuals with diseases^[1]^ or adopting a non-westernized lifestyle^[28]^, the differential prevalence of most *Bifidobacterium* species depending on age^[10]^, the high prevalence of *B. longum* throughout the human lifespan^[27]^, and the higher prevalence of *B. adolescentis* in healthy subjects than in those with diseases^[29,30]^.

We then studied the variability of *Bifidobacterium* community composition with the Dirichlet multinomial mixtures (DMM) method, which has been used for gut microbiome clustering on the basis of composition in many studies^[24,37-42]^. We identified partitions enriched in different combinations of *Bifidobacterium* species in a database containing predominantly adult data. The partitions that were more prevalent were characterized by a higher abundance of *B. longum* and *B. adolescentis,* whereas the partition corresponding to the non-detection of *Bifidobacterium* was the least prevalent. A specific analysis of three individual cohorts of adults (~1,000 subjects) confirmed the detection of several partitions, indicating an effect of between-subject variability rather than technical differences between studies. Those associated with a healthier state were dominated by *B. longum* and *B. adolescentis.* In previous studies, the species-level analysis revealed a positive correlation or covariation between multiple *Bifidobacterium* species^[43,44]^ or between specific species, such as *B. adolescentis* and *B. longum*^[45-47]^ or *B. adolescentis* and *B. bifidum*^[45]^. However, another study reported a negative correlation between B. adolescentis and B. longum^[48]^, suggesting variability between studies or study subjects. It remains unclear whether positive correlations indicate metabolic cross-feeding or similar niches, and this aspect requires further investigation *in vitro*. Cross-feeding between *Bifidobacterium* species on human milk oligosaccharides has been studied for the species prevalent in infants^[49-51]^, but*,* to our knowledge, there have been no studies investigating cross-feeding on complex dietary fibers between *B. longum*, *B. adolescentis* and *B. pseudocatenulatum*, which are more common in adults.

The partitions with a higher abundance of *Bifidobacterium*, and particularly those dominated by both *B. longum* and *B. adolescentis,* were associated with a higher gut microbiome diversity and a higher abundance of butyrate-producing species, including *Roseburia faecis, Coprococcus catus, C. eutactus*, *C. comes,* and *Eubacterium hallii*. Covariation between *Bifidobacterium* species and other resident species has been detected for butyrate producers in the metagenomic analysis^[43,46]^, and metabolic interactions between *B. adolescentis, B. longum,* and butyrate producers have been observed *in vitro* in the presence of complex dietary substrates^[52-54]^. These partitions were more frequently found in healthy subjects, suggesting that individual stratification exclusively on the basis of gut *Bifidobacterium* species abundance is associated with differential gut microbiome structure and state of health. Notably, the partition corresponding to an absence of *Bifidobacterium* detection, which contained a larger number of subjects with a non-westernized lifestyle than the other partitions, was depleted of *Streptococcus salivarius*, which is detected in consumers of yogurts, including yogurts supplemented with *B. animalis* subsp. *lactis*^[55]^. Overall, our results extend previous findings on associations with the gut microbiome by differentiating gut microbiomes enriched in specific *Bifidobacterium* types. It would be interesting to determine whether these partitions are associated with differential gut microbiome permissivity to distinct exogenous *Bifidobacterium* species/strains (during and/or after the cessation of consumption)^[56-58]^.

Following our exploratory analysis of the ecology of the *Bifidobacterium* community, we evaluated the functions of *Bifidobacterium* associated with partitions in a pangenomic analysis (i.e., functional variability within species). We studied *Bifidobacterium*-assigned MAGs retrieved from an extensive dataset for the human gut microbiome^[16]^, which could be assigned to metadata and gut microbiome features. EggNOG analysis confirmed known functional differences between the most prevalent *Bifidobacterium* species, such as the specificity of alpha-L-arabinofuranosidase to *B. longum*, involved in the metabolism of arabinans, arabinoxylans, and arabinogalactans^[27,59]^, glycoside hydrolases of host carbohydrate metabolism (mucin, human milk oligosaccharide) for *B. bifidum*^[60]^, and glycoside hydrolase GH 43 for *B. pseudocatenulatum*^[61]^. Asparagine synthetase was found to be highly specific to *B. adolescentis*^[62]^.

**Figure 5:**
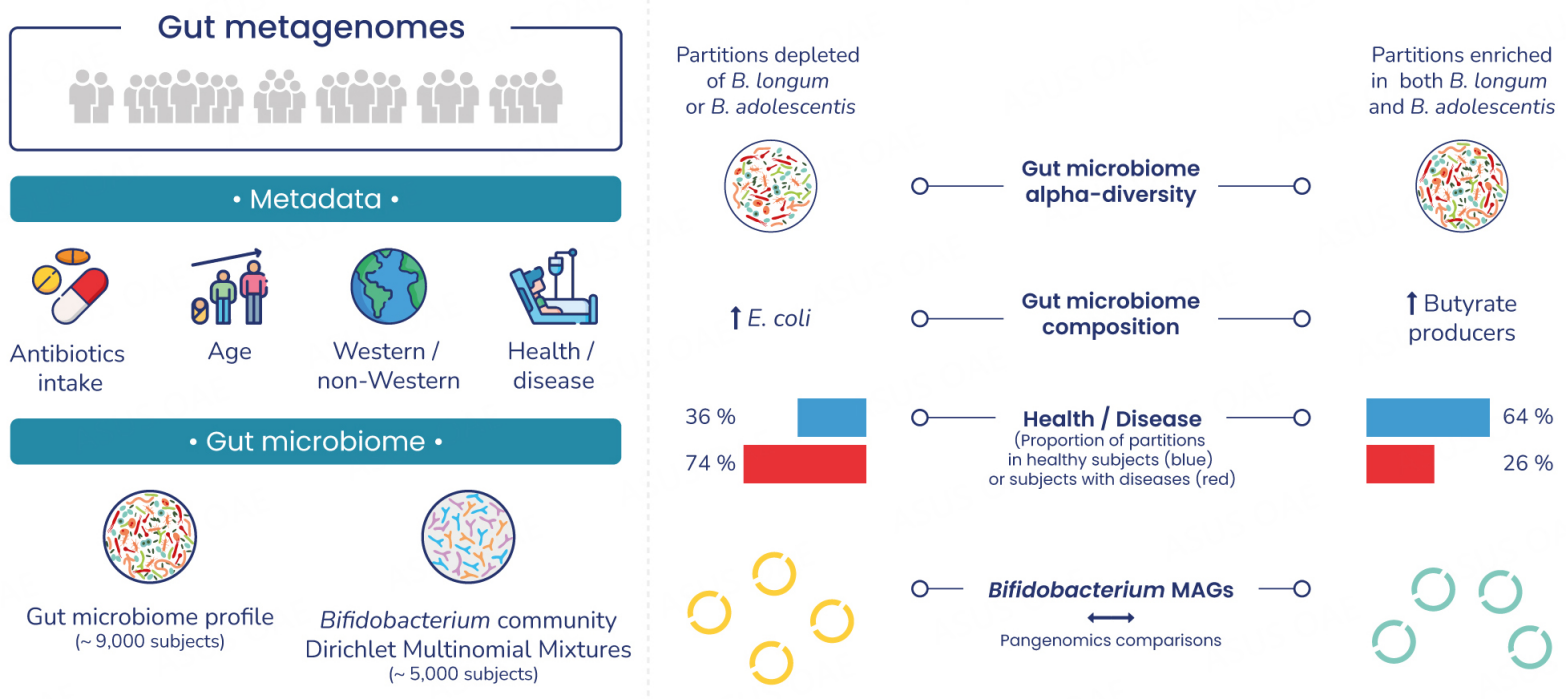
Graphical summary.

The association between species function and *Bifidobacterium* partitions revealed a difference in the functional features of *B. bifidum* MAGs across *Bifidobacterium* partitions in association with health status. Specifically, *B. bifidum* MAGs harboring a set of genes potentially related to phages were more prevalent in partitions associated with a lower gut microbiome diversity and were genetically more closely related. This potentially highlights the existence of a *B. bifidum* subspecies with a selective advantage for the colonization of gut microbiomes with a particular composition. Interest in the possible contribution of phages to gut microbiome ecology has increased significantly over the last decade, and one recent study^[63]^ showed the phages of *Bifidobacterium* to be rather specific. Overall, our pangenomic analysis revealed several functional features of *B. bifidum* differing between *Bifidobacterium* partitions as a function of health status [Figure 5].

This study has several limitations. First, it is based on the pooling of studies, an approach that is increasingly used to increase the sample size for ecological analysis. However, there are inherent differences in technical parameters between studies. Second, only a small amount of metadata is included. Diet is a major factor underlying gut microbiome variation between subjects. Carbohydrates are the dietary component most frequently reported to be positively associated with *Bifidobacterium*^[10]^. In previous metagenomics-based studies with species-level analysis, *B. adolescentis* was identified as the bifidobacterial species most significantly associated with dietary habits^[11]^, whereas both common and different associations between different *Bifidobacterium* species and food scores were identified^[12]^. The associations between dietary habits, partitions, variation of the LCT gene (lactase persistence), and other parameters should, therefore, be studied specifically in future studies. Another limitation of this study is that only cross-sectional analysis was performed. However, a previous metagenomic analysis revealed that the B. longum, B. adolescentis, and B. bifidum communities remained stable within individuals over a period of several years^[64]^, consistent with the stability of the genus *Bifidobacterium* reported in a 10-year study^[65]^.

This study is novel in the stratification of the gut microbiome according to specific resident bacterial species and the association with ecological features of the gut microbiome and health. These features could be applied to other genera. This first such study may provide insights for further investigations of the association between partitions and more exhaustive analyses of the host and environmental factors, including dietary habits. This exploratory study constitutes a first step towards understanding the ecology and variability of *Bifidobacterium,* with a view to guiding the selection of specific *Bifidobacterium* strains for use in subjects as a function of the partition present.

## DECLARATIONS

### Availability of data and materials

All the data used in this study are publicly available. CuratedMetagenomicData is available from https://github.com/waldronlab/curatedMetagenomicData.

### Copyright

© The Author(s) 2023.
