# Supplementary material for: Exploring Bifidobacterium species community and functional variations with human gut microbiome structure and health beyond infancy
Source: Microbiome Res Rep. 2023 Mar 31;2(2):9. doi: 10.20517/mrr.2023.01 (PMC10688807; doi:10.20517/mrr.2023.01)
Supplement: Supplementary file 1 [file mrr-2-2-9-SupplementaryMaterials.zip › SupplementaryMaterials.pdf]

SUPPLEMENTARY FIGURES

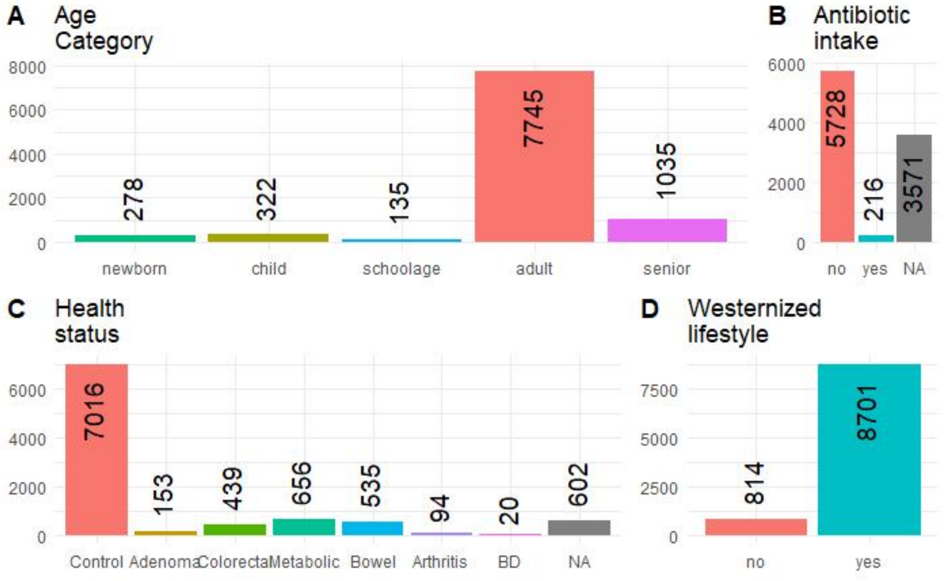

Supplementary Figure 1. Number of subjects per type of metadata. (A) Age category (B) Antibiotic intake (C) Health status (D) Westernized lifestyle NA: data not available.

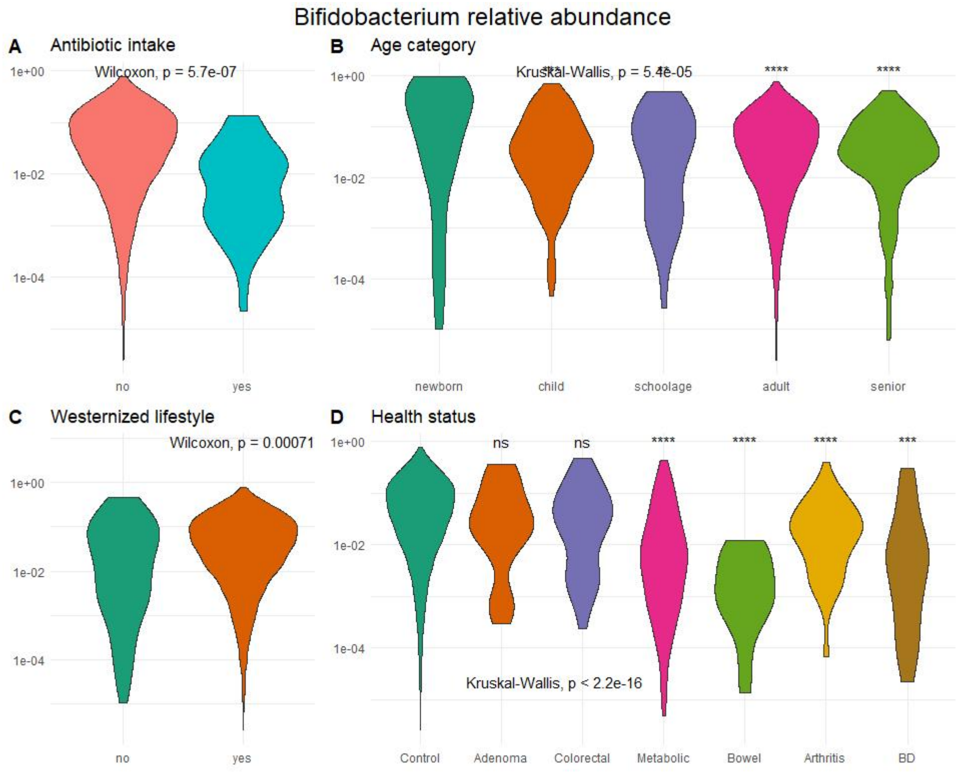

Supplementary Figure 2. Relative abundance of *Bifidobacterium* as a function of (A) Antibiotic intake, (B) Age-category without recent antibiotic intake (C) Westernized lifestyle in adults without recent antibiotic intake, (D) Health status in adults without recent antibiotic intake. Kruskal-Wallis tests (A and D) and Mann-Whitney test against the controls: no (B and C), newborn (A), and control (D). ns: not significant; $**P < 0.01$; $***P < 0.001$; $****P < 0.0001$". See Table S1 for number of subjects. BD: Behcet's disease.

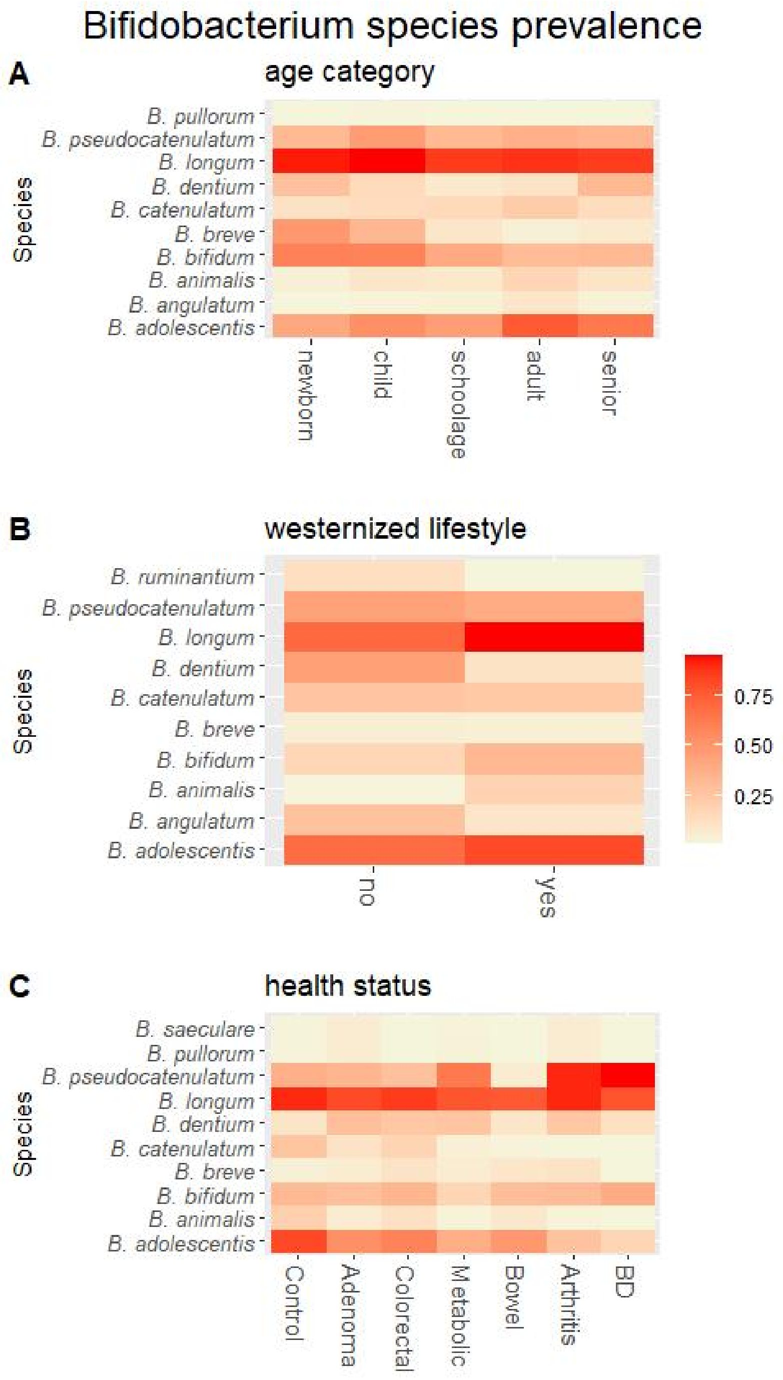

Supplementary Figure 3. Heatmap of the 10 most prevalent *Bifidobacterium* species as a function of (A) Age category for subjects with no antibiotics, (B) Westernized lifestyle on adults with no antibiotics and (C) Health status on adults with no antibiotics BD: Behcet's disease.

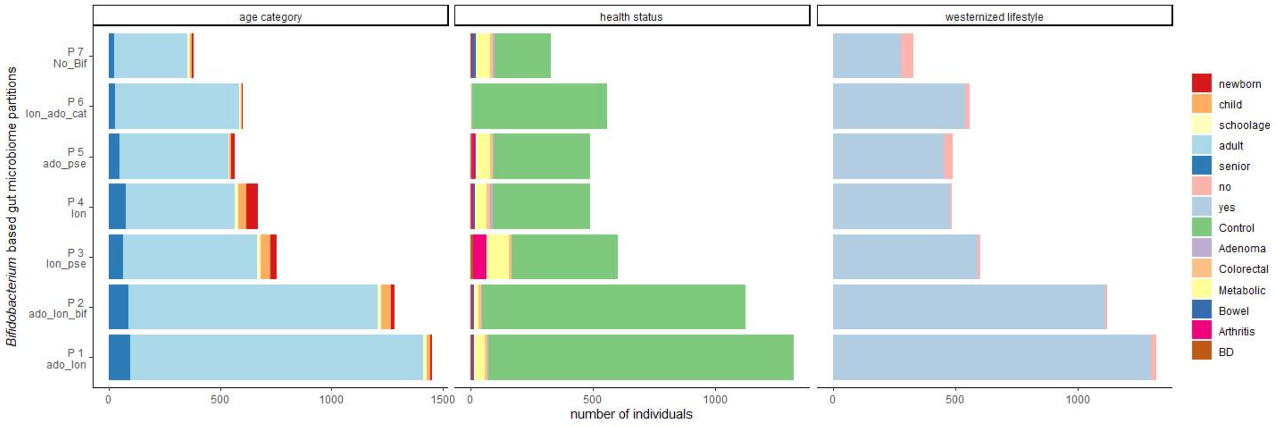

Supplementary Figure 4. Number of subjects between *Bifidobacterium*-based gut microbiome partitions according to metadata (without recent antibiotic intake). (A) Age category (B) Health status (adults) (C) Westernized lifestyle (adults).

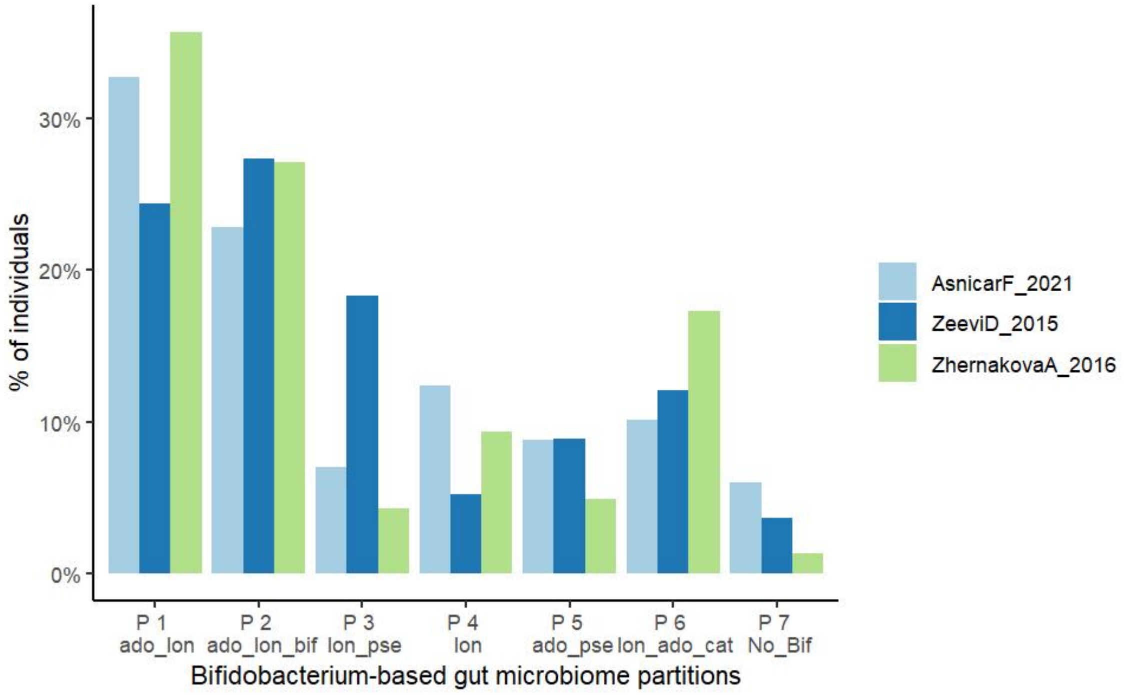

Supplementary Figure 5. Prevalence of *Bifidobacterium*-based gut microbiome partitions in three large cohorts of adults integrated into the CMD (Asnicar *et al.*, *N* = 1,098), Zeevi *et al.* (*N* = 800), Zhernakova *et al.* (*N* = 1,135).

SUPPORTING TABLES

Supplementary Table 1. Main features of *Bifidobacterium*-based partitions (number of reads, relative abundance of *Bifidobacterium,* and Shannon index).

Supplementary Table 2. OGs associated with each *Bifidobacterium* species.

Supplementary Table 3. OGs associated with health-*Bifidobacterium* partitions. 15 OGs related to phages (integrases, transposases, and helicases) are in bold.
